# Viability of Preictal High-Frequency Oscillation Rates as a Biomarker for Seizure Prediction

**DOI:** 10.3389/fnhum.2020.612899

**Published:** 2021-01-28

**Authors:** Jared M. Scott, Stephen V. Gliske, Levin Kuhlmann, William C. Stacey

**Affiliations:** ^1^Department of Biomedical Engineering, University of Michigan, Ann Arbor, MI, United States; ^2^Biointerfaces Institute, University of Michigan, Ann Arbor, MI, United States; ^3^Department of Neurology, University of Michigan Hospitals, Ann Arbor, MI, United States; ^4^Department of Neurosurgery, University of Nebraska Medical Center, Omaha, NE, United States; ^5^Department of Data Science and AI, Faculty of Information Technology, Monash University, Clayton, VIC, Australia

**Keywords:** epilepsy, seizure prediction, preictal identification, high frequency oscillation, ROC analysis

## Abstract

**Motivation:** There is an ongoing search for definitive and reliable biomarkers to forecast or predict imminent seizure onset, but to date most research has been limited to EEG with sampling rates <1,000 Hz. High-frequency oscillations (HFOs) have gained acceptance as an indicator of epileptic tissue, but few have investigated the temporal properties of HFOs or their potential role as a predictor in seizure prediction. Here we evaluate time-varying trends in preictal HFO rates as a potential biomarker of seizure prediction.

**Methods:** HFOs were identified for all interictal and preictal periods with a validated automated detector in 27 patients who underwent intracranial EEG monitoring. We used LASSO logistic regression with several features of the HFO rate to distinguish preictal from interictal periods in each individual. We then tested these models with held-out data and evaluated their performance with the area-under-the-curve (AUC) of their receiver-operating curve (ROC). Finally, we assessed the significance of these results using non-parametric statistical tests.

**Results:** There was variability in the ability of HFOs to discern preictal from interictal states across our cohort. We identified a subset of 10 patients in whom the presence of the preictal state could be successfully predicted better than chance. For some of these individuals, average AUC in the held-out data reached higher than 0.80, which suggests that HFO rates can significantly differentiate preictal and interictal periods for certain patients.

**Significance:** These findings show that temporal trends in HFO rate can predict the preictal state better than random chance in some individuals. Such promising results indicate that future prediction efforts would benefit from the inclusion of high-frequency information in their predictive models and technological architecture.

## Introduction

One of the most debilitating aspects of epilepsy is the uncertainty patients feel, not knowing when the next seizure will occur. Though seizures themselves account for an extremely small percentage of an individual's time (Cook et al., 2013), the constant threat of a seizure can make the planning of normal day-to-day activities an impossibility for some (Bishop and Allen, 2003). This has led many investigators to search for methods to predict when seizure might occur (Mormann et al., 2005; Freestone et al., 2015, 2017; Gadhoumi et al., 2016; Kuhlmann et al., 2018a).

While “seizure prediction” has been an attractive research subject for decades, early efforts had many unforeseen challenges. While there was evidence that EEG changed in the minutes or hours before seizures (Mormann et al., 2005), it was difficult to prove that these measures could work prospectively. A major breakthrough occurred when rigorous statistics were developed—the key was to show that a given algorithm could outperform random chance (Mormann et al., 2007; Snyder et al., 2008). Several studies then followed using this method and were able to show that intracranial EEG signals could predict the preictal state better than chance (Cook et al., 2013; Karoly et al., 2017; Kuhlmann et al., 2018b). Critical in that work was the unprecedented collection of months of continuous EEG in a clinical trial in Australia, which allowed for rigorous long-term statistics (Cook et al., 2013; Kuhlmann et al., 2018b). That dataset has become a crucial tool in later work, including international competitions (Kuhlmann et al., 2018b), as prediction algorithms have made many further improvements (Alexandre Teixeira et al., 2014; Karoly et al., 2017; Truong et al., 2018; Stojanović et al., 2020). However, the data also have two important limitations: the data were acquired at low sampling rate (200 Hz) that does not allow analysis of high-resolution EEG signals, and more importantly, since the trial ended no similar chronic recordings have been collected.

Thus, while there have been many very promising results in the field of seizure prediction, most work has been focused on a single dataset of long-term, low-resolution intracranial EEG. The results have proven that seizure prediction is possible in many patients but clearly are far from optimal. One potential avenue for further improvement is the possibility that higher-resolution EEG could hold greater information. In particular, over the past 20 years it has become increasingly apparent that high-frequency oscillations (HFOs) are a powerful biomarker of epilepsy (Jacobs et al., 2012; Zijlmans et al., 2012; Frauscher et al., 2017; Jacobs and Zijlmans, 2020). HFOs consist of short (<100 ms) oscillations in the 80–500-Hz frequency band and require sampling rates of at least 2,000 Hz for accurate identification (Gliske et al., 2016a). HFOs are more likely to occur in the epileptogenic zone (Jacobs et al., 2012) and may help guide surgical decisions (Cho et al., 2014; Höller et al., 2015; Fedele et al., 2017; van 't Klooster et al., 2017). One relatively unexplored aspect of HFOs is that their characteristics can also change in the 30 min prior to seizure initiation in certain individuals (Jacobs et al., 2009; Pearce et al., 2013). These preliminary studies were constrained by small patient cohorts and datasets that were not as specific as currently available methods (Blanco et al., 2010, 2011). Nevertheless, the evidence from those studies motivate using HFOs to identify the preictal state.

Utilizing population-level inference and a large clinical dataset, our group recently found several features of HFO rates that were highly correlated with the preictal state (Scott et al., 2020). In that work, we averaged the HFO response over all available data per patient and compared the responses during interictal and preictal epochs; several patients had significant results. However, in order to utilize HFOs to identify the preictal state prospectively, a different analysis is necessary. The HFO response in a given segment of data must be compared individually to that of other segments, rather than in aggregate as in that prior work.

Robust implementation of seizure detection algorithms requires several months of continuous recording, as was accomplished by the Neurovista trial in Australia (Cook et al., 2013). Such data with a sufficient sampling rate to detect HFOs is currently impossible to attain. Until such devices are available, the only alternative is to utilize inpatient intracranial EEG monitoring, which lasts <2 weeks. Although such data are vastly inferior, they are also the only current option. Until implantable devices with >1,000 Hz sampling rate are available, the role of HFOs in the specific context of seizure prediction must first be evaluated using only the limited intracranial monitoring data available, which is our goal herein.

With this study, we evaluate the preliminary usefulness of HFOs in patient-specific seizure prediction. We employ state-of-the-art automated HFO detection methods on the entire recorded intracranial EEG data of a clinically diverse cohort of 27 patients. With more than 10 million detected HFOs in this dataset, we use various features of HFO rates as predictors in patient-specific preictal classification models. With robust machine learning methods and statistical techniques to validate our results, we find that 10/27 patients have excellent classifier performance. These results are limited due to the short recording periods but were very promising. While the technology does not yet exist that would allow a full prospective analysis using high-resolution data, these results motivate future studies that incorporate such technology in the next generation of seizure prediction devices.

## Methods

### Patient Population

To form our patient cohort, we looked at all patients with refractory epilepsy who had undergone intracranial EEG (iEEG) monitoring at the University of Michigan from 2016 to 2018. In order to ensure that sufficient data was available for training and testing our models, we required patients with the following: (1) a defined seizure onset zone, (2) at least three recorded seizures that were each preceded by non-zero HFO rates, and (3) the availability of at least 24 h of data; applying these criteria to the 32 available patients resulted in 27 patients. The study was approved by the local IRB, and all patients in the study consented to have their EEG data de-identified for later analysis. Of note, all data were acquired under standard clinical procedures, and the current work was done retrospectively: no data from this research had any effect on the clinical care. Further summary of the patient population is found in Table 1.

**Table 1:** Clinical data.

**Subject**	**Age**	**Sex**	**ILAE outcome**	**Seizure focus (hemisphere, region)**	**Pathology/*implant type***	**Number of intracranial channels**				**Total recorded time (hours)**	**Total number HFOs**	**Mean HFO rate (#/min./channel)**		**Number of seizures**				**Responder window subset (window duration, min.)**		
						**Total**	**ECoG**	**depth**	**SOZ**			**SOZ**	**OUT**	**Total**	**Used**	**Training**	**Testing**	**30**	**15**	**10**
UMHS-0018	41	M	Ib	L F	CD	32	0	32	4	59.8	108,510	4.18	0.54	3	3	2	1			
UMHS-0019	59	F	II	R T	Gliosis	106	106	0	2	168.8	170,946	2.30	0.19	5	3	2	1			
UMHS-0020	45	F	II	R T	MTS	25	0	25	9	171.2	54,254	0.38	0.12	7	7	5	2			
UMHS-0021	30	M	II	R T	Gliosis, PVNH, PMG	46	0	46	13	179.5	394,398	1.98	0.50	9	7	5	2			
UMHS-0023	29	M	NR	L T, P	PVNH/*Neuropace*	69	41	28	29	164.3	390,134	0.86	0.37	20	9	6	3			
UMHS-0024	31	M	NR	L, R T	*Neuropace*	75	55	20	16	177.2	1,649,380	3.40	1.71	28	11	7	4			
UMHS-0025	17	F	II	L T	Gliosis	20	0	20	5	207.7	270,125	1.75	0.86	10	5	3	2			
UMHS-0026	22	F	NR	R T	PVNH	52	0	52	3	246.2	382,201	1.28	0.45	40	10	7	3	X	X	X
UMHS-0027	26	M	NR	L Diffuse	*VNS*	91	81	10	3	205.2	1,601,359	1.90	1.41	97	11	7	4			
UMHS-0028	14	F	I	R T	Tumor: Glioma	53	47	6	5	79.7	140,782	2.95	0.42	7	6	4	2	X	X	X
UMHS-0029	48	M	NR	L T, Occ.	*Neuropace*	91	91	0	22	226.3	847,560	0.60	0.71	14	7	5	2			
UMHS-0030	5	M	III	L T	MTS, Gliosis	100	100	0	2	146	330,614	0.98	0.56	33	21	14	7	X		X
UMHS-0031	13	M	I	L T	Gliosis, Tumor: NF1	99	99	0	6	180	263,676	1.17	0.39	9	4	3	1			
UMHS-0032	41	F	I	R F	CD	32	0	32	3	184.3	295,865	3.79	0.96	8	6	4	2			
UMHS-0033	5	F	II	R Ins.	CD, Gliosis	74	0	74	4	120.7	233,883	1.40	0.38	28	8	5	3		X	X
UMHS-0034	33	F	I	R F	Gliosis	32	0	32	11	136.3	448,718	2.58	1.26	17	16	11	5	X		
UMHS-0035	50	F	I	L T	Gliosis	57	57	0	2	162.7	108,147	0.73	0.21	7	4	3	1		X	
UMHS-0036	43	M	NR	L, R T	CD/*Neuropace*	54	0	54	2	172.5	347,928	1.34	0.60	18	12	8	4			
UMHS-0039	47	M	NR	R P	CD/*Neuropace*	90	0	90	10	155.2	266,422	1.02	0.23	19	9	6	3			
UMHS-0040	14	F	I	L P	CD, Gliosis	63	55	8	8	196.7	323,180	0.38	0.66	7	7	5	2		X	
UMHS-0041	32	F	I	R F	CD	71	0	71	9	176.5	43,350	0.27	0.04	36	3	2	1			
UMHS-0043	28	M	II	R T	Gliosis	86	0	86	9	182.2	386,967	1.34	0.42	46	16	11	5		X	X
UMHS-0044	45	F	NR	L T, P	*Neuropace*	76	0	76	6	170.2	414,195	1.29	0.47	13	5	3	2			
UMHS-0045	17	F	NR	L, R T	*Neuropace*	94	0	94	15	331.5	631,551	0.79	0.25	6	6	4	2		X	
UMHS-0046	23	F	I	L F	CD	30	0	30	9	139.3	16,575	0.15	0.04	17	5	3	2			
UMHS-0048	22	F	NR	L, R T	*Neuropace*	86	0	86	8	141.8	404,972	2.76	0.33	23	8	5	3	X	X	X
UMHS-0049	53	F	NR	L, R T	*Neuropace*	94	0	94	15	176.8	287,303	0.98	0.16	17	5	3	2			
					**Totals**/**averages**	1,798	732	1,066	230	4658.6	10,812,995	1.58	0.53	544	214	143	71	5	8	6
										172.5	400,481			20	8	5	3			

*Number of unique responders: 10 (37%)*.

*M/F, male, female; L/R, left/right; T, temporal; P, parietal; F, frontal; Occ, occipital; NR, not resected; CD, cortical dysplasia; MTS, medial temporal sclerosis; PVNH, periventricular nodular heterotopia; PMG, polymicrogyria; VNS, vagal nerve stimulator; DNET, dysembryoplastic neuroepithelial tumor; NF1, neurofibromatosis type 1*.

### Data Acquisition

All intracranial recordings were sampled at 4,096 Hz with a Quantum amplifier (Natus Medical Inc., Pleasanton, CA); the electrodes implanted for monitoring consisted of subdural grid, depth, and stereo-EEG electrodes, as deemed appropriate for each patient during standard clinical care. All recordings were referenced to a lab-standard instrument reference placed midway between Fz and Cz when first recorded and then were re-referenced for HFO detection using common average referencing (Gliske et al., 2016b), which was applied to all electrodes of the same type, e.g., all depths or all grids or strips together. The treating epileptologist determined which channels comprised the seizure onset zone (SOZ channels), as well as the onset and offset times of all seizures; we obtained these metadata through the official clinical report for a given patient. Channels within the resected volume of tissue (RV channels) were identified and labeled through consultation with the neurosurgeon and by pre- and post-op imaging comparisons if available. Any channel that was not labeled as an SOZ or RV channel was labeled as an OUT channel. Note that a seizure prediction algorithm should have knowledge of the SOZ and OUT channels available, as it must be trained on previous seizures and would be implemented after these studies are completed. It is also important to note that the SOZ is what was determined by the reading clinician and does not depend upon being the true epileptogenic zone. We incorporated the analysis of OUT channels as a conservative way to account for diagnostic uncertainty and see if other channels also had useful information. Channels labeled as RV that did not overlap with the SOZ were not used in our analysis, in order to maintain a more conservative analysis.

### Data Analysis

**Figure 1:**
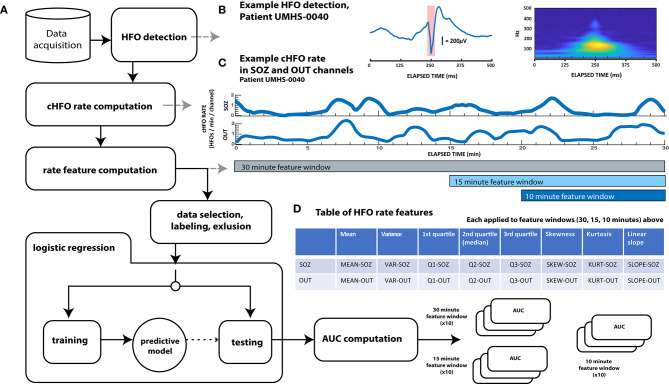
Schematic diagram showing overall data analysis workflow. **(A)** General analysis workflow. After automated HFO detection, continuous HFO rates (cHFO rate) are computed in both the SOZ and OUT channel groups. Next, several statistical quantities (features of HFO rate) are computed from cHFO rates in three “feature windows” of different durations: 30-, 15-, and 10-min feature windows. After labeling this feature data as either preictal or interictal, observations that remain after an exclusion process are randomly divided into training and test data sets. Training data is used to train predictive LASSO logistic regression models, which are then tested with unseen testing data. The performance of each model with this testing data is assessed by computing the test AUC value, which, when averaged over 10× cross-validation runs for each of the three feature windows, are finally compared across patients; these results are visualized in Figure 3. **(B)** Example HFO detection, “responder” patient UMHS-0040. The HFO waveform is displayed on the left, while its time–frequency decomposition (computed with the Morse wavelet) is visualized on the right. **(C)** Example of cHFO rates computed for patient UMHS-0040. Continuous HFO rates (cHFO rate—defined as HFOs/min/channel) are computed in both the SOZ and OUT channel groups separately. The rate features used in the proceeding Table **(D)** are computed from these cHFO trajectories in 30-, 15-, and 10-min segments. **(D)** Table of rate features. Eight features are applied to cHFO rates per channel group (SOZ and OUT channel groups), which yields a total of 16 rate features. Abbreviations shown in this table are used throughout the text.

All data analysis was conducted with custom MATLAB (Mathworks, Natick, MA) and C++ functions and scripts. As described in detail below and shown in the block process diagram of Figure 1, this analysis consisted of several steps: first, automated HFO detection was performed on all patient data. Then, several features across consecutive time windows of varying duration were computed from HFO rates. These features were used to train a logistic regression model to distinguish preictal vs. interictal states. The algorithm was cross validated with held-out data and compared vs. random chance. Model performance was quantified using ROC curves.

### Automated HFO Detection

All HFOs were identified with a validated automated detector (Gliske et al., 2016b) with additional modifications described further below. In summary, this detector is based upon the original “Staba” RMS-based detector (Staba et al., 2002) which then increases the specificity by redacting detections that overlap in time with several EEG artifacts such as sharp transients, electrical interference and noise, and artifacts from signal filtering. To further increase HFO specificity, we excluded detected events with waveforms consistent with features of muscle (EMG) artifact, using another validated algorithm (Ren et al., 2019) as in our previous work (Scott et al., 2020). Of note, these algorithms have previously been shown to be similar to human reviewers (Gliske et al., 2016b, 2020).

We also modified the data processing pipeline of our automated detector to ensure that it functioned appropriately within the unique constraints of seizure prediction. Most automated detectors operate by processing incoming EEG data in successive epochs of fixed length, e.g., 10 min, and then assess the background activity of the entire epoch to determine a threshold for detecting HFOs within that epoch. That process cannot happen in real-time nor (pseudo)prospectively, because evaluating a potential HFO at a specific point in time requires knowledge of background activity that has yet to occur. Such a process would not be possible for prospective seizure prediction, in which there should be no knowledge of the future. To address this constraint, we modified the detection algorithm to work prospectively. First, we approximated real-time detection by only detecting HFOs for 30 s at time. Second, we still used 10 min of EEG to calculate the background, but use the *previous* 10 min of EEG data, relative to the end of each of data segment. In effect, the algorithm is identical to the previous one except it only reports the HFOs that are detected during the final 30 s of a 10 min segment, and the same process is repeated by sliding the 10-min window forward 30 s. One outcome of this is that the first HFOs detected in any given data file start after the first 10 min of recording. With these adaptations, our automated HFO detection was better suited to the constraints of seizure prediction and more closely resembled a real-time process. Further—and perhaps most importantly for preictal HFO detection—these changes also prevented seizure activity from influencing the detector (see section Feature Data Labeling and Exclusion). We compared these results to those of the original detector, and there was no appreciable difference in HFO rate (data not shown), which is expected since there were no changes inherent to the detector itself, but rather how it was fed data.

### Computation of HFO Rate

In order to investigate temporal variations in HFO rate with sufficient resolution, we approximated HFO rate (which we define as the number of HFOs per minute per channel) in both SOZ and OUT channel groups as a continuous function of time (cHFO rate). The cHFO rate was obtained by calculating the estimated HFO rate during 1 min of data, then sliding the 1-min window forward 1 s and recalculating. This sliding window method approximates a continuous HFO rate with a 1-s time resolution. The sliding window was applied to all SOZ or OUT channels, which were grouped separately. For a given window segment and channel group, the HFO rate was computed by summing the number of HFOs occurring across all channels of the same group; this value was then divided by the total number of channels in that respective group, which resulted in an estimate of the average cHFO per channel within each group (SOZ or OUT).

### Features of HFO Rate

The advantage to using cHFO rate as computed above—rather than averaging it over longer periods—is that the temporal resolution of cHFO rates can reveal fluctuations and patterns in HFOs down to the scale of a second—which could be important in characterizing preictal trends. We quantified the temporal variation of cHFO rates with several descriptive statistics, including mean, variance, linear slope, quartiles, skewness, and kurtosis across a given epoch of time. We also compared linear trends in cHFO rates using the slope extracted from linear regression applied to cHFO rates for a given epoch of time. All these values were computed separately in SOZ and OUT channel groups across three different epochs of time: 30, 15, and 10 min, which we call “feature windows.” The feature windows were designed to account for possible differences in seizure horizons between patients, as we hypothesized that the duration of any preictal state would not be constant across the entire cohort. All features were computed from the start of a given data file in consecutive 1-min intervals. Each feature window was analyzed independently of the others throughout the entirety of the study.

### Feature Data Labeling and Exclusion

In machine learning, classification algorithms used in prediction need labeled observations of data in order to train their models. In this case, we label data as either interictal or preictal. Based on our prior data showing HFO features changing up to 30 min prior to seizures (Pearce et al., 2013; Scott et al., 2020), we defined the “preictal period” as the 31 min prior to the start of the seizure. The extra minute occurs because we inserted a buffer of 1 min just prior to seizure onset, which accounts for some interrater variability in seizure onset time (Abend et al., 2011).

For each of the feature windows (10, 15, or 30 min), the “preictal” windows were defined as the last window immediately prior to the seizure, but not including any of the 1 min just before seizure onset. Because the calculations slide forward in 1-min steps, this means each “preictal” feature window ends between 1 and 2 min prior to the clinician-determined seizure onset time. For each feature window length, we only included the one “preictal” window immediately before the seizure. Because our prior data suggested up to 30 min could be considered as the physiological preictal period, to be conservative we ignored data during that period that was not in the “preictal” feature window. Data from those times (the two previous 10-min windows and one previous 15-min window) were discarded from both the preictal and interictal analysis.

“Interictal” was defined as all data starting 11 min after a seizure until 31 min prior to the next seizure, which allows a 1-min buffer for uncertainties in the start/stop times of the seizure. We note that some research has shown that the preictal state may extend beyond 30 min (Litt et al., 2001; Stacey et al., 2011), so this definition is conservative and may not capture all differences. We calculated an “interictal” feature window for every consecutive epoch (i.e., every 30 min for the 30-min feature window; every 10 min for the 10-min feature window).

There were other limited circumstances that we excluded from analysis. To ensure that seizures were evaluated independently of other seizures, such as when multiple seizures occur sequentially, we redacted preictal observations falling within peri-ictal extent (11 min postictal or 31 min preictal) of other seizures. Further, we also redacted any observation that overlapped with periods of incomplete or missing data, which could result from gaps within a file or from a file's end. Finally, considering our modifications to the HFO detector, any data observation overlapping with the first 10 min of a given data file was also redacted, as HFOs are not detected for the first 10 min.

### Logistic Regression Model

We used a logistic regression model to classify preictal vs. interictal data. Logistic regression determines the probability that given data is from a specific labeled class and has been used in seizure prediction studies previously (Mirowski et al., 2009). It also has the advantage of allowing us to analyze the relative contributions of each feature, rather than being a “black box” approach. We trained models for each of the three feature windows (10, 15, 30 min) using 2/3 of the data and then testing on the remaining 1/3. This process was cross-validated 10 times for each feature window by randomly selecting different interictal and preictal data, and re-running the training and testing step, for a total of 30 models per patient. Random selection, rather than chronological, was used because of the limitations of this dataset: unlike in the Neurovista dataset that had months for the recordings to stabilize (Ung et al., 2017), our data is limited to 2 weeks of inpatient monitoring. This unavoidably leads to some variability over time due to various factors such as medication taper, sleep disturbances, and the settling of electrodes (Zijlmans et al., 2009; Ung et al., 2017; Gliske et al., 2018). Here, we used random selection to reduce the influence of these factors on overall model performance, but this also may reduce the effectiveness of the model.

In order to facilitate the models helping to determine which coefficients were most useful in forecasting seizures, we used LASSO logistic regression (Mirowski et al., 2009; Tibshirani, 2011; Lu et al., 2020) to create the predictive models used in our study. Specifically, in Matlab we used the lassoglm function, with the following general syntax: lassoglm(XTrain, yTrain, “binomial,” “CV,” k), where XTrain is the feature vector, yTrain is a binary vector with “0” for interictal and “1” for preictal, and k is chosen as the number of seizures within the training data. This function inherently cross-validates the trained model based upon the number of seizures k, which reduces overfitting. In general, LASSO introduces a penalty on the absolute value of the coefficients, and optimizes the model by iterating through different penalty parameters to find the lowest error, while removing coefficients that have minimal effects (Tibshirani, 2011). Thus, one outcome of the training step is to identify which features were the most important for identification of the preictal state.

### Assessing Predictive Performance

Each cross-validation iteration tests whether the predictive model can correctly classify novel preictal vs. interictal data. We computed the ROC curve for each iteration, then computed the arithmetic mean of all the areas under the curve (AUC) across all 10 iterations. A random predictor would have an AUC of 0.5, while a successful predictor should have an AUC higher than 0.5. We chose a nominal threshold of 0.6 to show the minimal improvement above 0.5 that would be meaningful. However, that threshold is subjective so we then tested the significance of each AUC using bootstrapping by randomizing preictal and interictal labels (*n* = 1,000). The statistical significance of these average AUC was determined by taking the harmonic mean of the bootstrap *p*-values (Wilson, 2019), a procedure used in meta-analysis to combine *p*-values from multiple tests. Successful tests were those in which the average AUC was ≥0.6 and *p* < 0.05. We note that in clinical practice an AUC of 0.6 might be difficult to implement successfully on its own; however, it is comparable with prior seizure prediction work in standard EEG (Mormann et al., 2005; Freestone et al., 2015, 2017; Gadhoumi et al., 2016; Kuhlmann et al., 2018a).

## Results

Our heterogeneous patient cohort was comprised of individuals with a variety of ages, clinical etiologies and pathologies, and seizure foci. Out of 32 original patients in our database, four patients (UMHS-0037,−0038,−0042,−0047) were excluded because of either insufficient recorded seizures or undefined seizure onset zones. One patient in particular (UMHS-0022) had seizures with no HFOs prior to onset; this patient was also excluded, which left a total of 27 patients remaining for further analysis. Across these 27 patients, we detected more than 10 million HFOs across over 190 total days of intracranial EEG recordings. Over 210 seizures and 3,800 h of interictal data (average of 8 seizures and 141 h per patient) were used to train and test our classification models.

### Comparison of Test AUC Values

**Figure 2:**
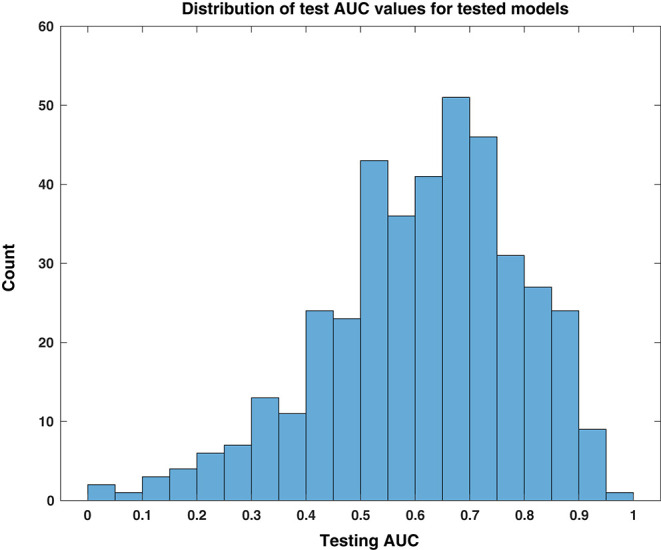
Distribution of test AUC values for tested models. This histogram of testing AUC values, computed for all tested models individually over all patients and feature windows, is skewed toward predictive performance that is better than random chance, i.e., values higher than 0.5.

We first assessed the general responses across all cross validation models in all patients. Over the 27 patients, with 30 models each (810 total), the model successfully converged to a solution in 403 instances (49.8%). The non-converging solutions are easily identified because all coefficients for HFO features are 0, and it is obvious that the model could not be used. In such cases, we conservatively assigned them a testing AUC value of 0.5 (and a bootstrap *p*-value equal to 1)—the same performance as a random predictor. The remaining patient models were composed of linear combinations of HFO rate features. As shown in the histogram of Figure 2, the distribution of test AUC values for these models overall showed significant variability and spread from 0.5 (AUC test—maximum: 0.97, minimum: 0.024, median: 0.64). The skew of this distribution toward values >0.5 suggests that a significant portion of models that used HFO features could perform better than random chance at identifying the preictal period.

**Figure 3:**
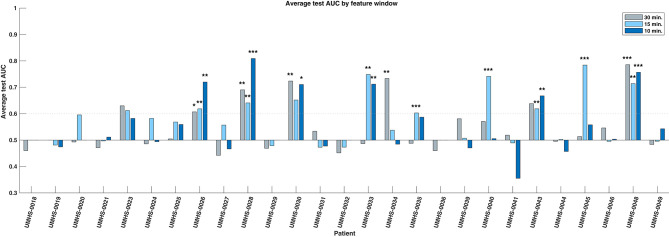
Bar chart of average test AUC values by patient and feature window. Ten individual responder patients have significant predictive performance (average test AUC >=0.6, significant average bootstrap test *p* < 0.05) in one or more feature windows. The statistical significance of the bootstrap test per feature window is indicated with asterisks: *p < 0.05, **p < 0.01, ***p < 0.001, respectively. Note that the significance is based upon how likely that patient's data could produce the given AUC by random chance, not whether the magnitude of the AUC itself is high.

We evaluated the consistency and reliability of this result within patients by determining if its average test AUC was at least 0.6 and if the average bootstrapped *p*-value was <0.05. These values are shown with statistical significance noted in the bar plots of Figure 3. We found that 10 out of the 27 patients had a significant response in at least one of the feature windows. We denote these 10 patients as “responders,” and their average predictive response was robust and consistent. The presence of this subset of patients in our cohort suggests that there are measurable changes in preictal HFO rate preceding epileptic seizures that deviate from interictal trends. This finding shows that HFOs can act as a temporal biomarker of seizure onset in some patients.

Within the responder group, 4 were significant in only one feature window, while the rest had multiple. We compared the three windows (10, 15, 30 min) and found no evidence that the performance of one window was better than any other—either by how frequently it was significant in these patients, or by how high its overall performance was (Chi-square test: *p* = 0.61; Kruskal–Wallis test: *p* = 0.737). All responders and their significant windows are identified in Figure 3 and in Table 1. The *p*-value and associated asterisks indicating statistical significance in Figure 3 were based on individual bootstrap tests and not corrected for multiple comparisons.

### Significance of Responder Predictors

**Figure 4:**
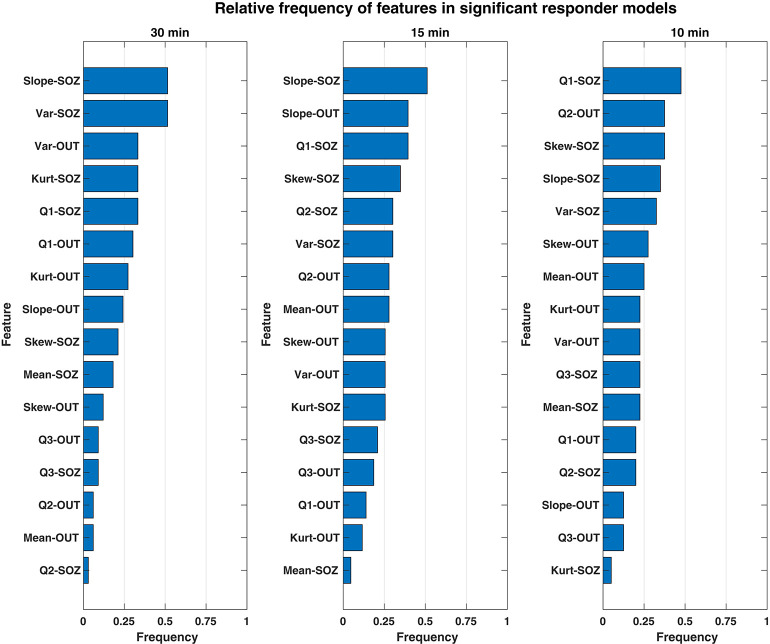
Bar chart showing the relative frequency of each rate feature for only significant responder models. The features of HFO rate most important to discerning the preictal HFO response in responders are ranked in descending order (top to bottom) according to how often their respective model coefficients were non-zero for a given feature window. Overall, the most important feature was the SLOPE-SOZ feature, which was ranked first in the both 30- and 15-min feature windows. Also important were features in OUT channels, a novel finding that suggests HFOs outside epileptic tissue could still be involved in the process of seizure generation.

We investigated which features contributed to the significant predictive response observed in responder patients. Overall, both the combination and relative magnitude of HFO features in responder models varied significantly between patients, feature windows, and even between different cross-validation runs. Considering this variability, we could not evaluate feature importance directly by the raw coefficient values that resulted from LASSO logistic regression. Instead, we calculated how often a given feature was included among models—specifically, how often its corresponding coefficient was non-zero. In this manner, we considered the most commonly used features to be the most important to differentiating the preictal state from other interictal observations—whether its associated output coefficient was positive (which would indicate increased likelihood of an imminent seizure resulting from an increase in the feature's value) or negative (i.e., decreased seizure likelihood from a feature's increase). These frequencies of non-zero model coefficients per feature are shown by a feature window in Figure 4 and are sorted in order from most to least common within responder models. Though we did not evaluate feature magnitude directly, we note that the medians of all responder SLOPE-SOZ features by patient and feature window were all positive, which reinforces our prior findings that gradually increasing HFO rates anticipate seizure onset (Scott et al., 2020).

While there were some observed differences in which features were the most common between window durations, there were no statistically significant differences in feature frequency across the three feature windows (Kruskal–Wallis: *p* = 0.64). In terms of the most important features, the linear slope of HFO rate in the SOZ (Slope-SOZ) was most important in both the 30- and 15-min windows. Also common among important features were those computed from cHFO rates in OUT channels—channels that might be traditionally considered as less involved in pathological brain networks. Yet, there were no statistical differences in frequency between SOZ and OUT channel features (rank-sum tests: *p* = 0.34, = 0.24, = 0.42 for 30-, 15-, and 10-min windows, respectively), even though SOZ features were highest ranked across feature windows, with an average cumulative frequency almost 14% greater than that of OUT channel features. This suggests that HFO rates could be used to identify the preictal state regardless of their location.

### Clinical Factors of Responders

Considering the clinical outcomes of responders, four were ILAE class I, two were class II, there was one class III, and the others were not resected. Comparing various clinical factors, there was no statistical evidence for differences in the composition of responder patients compared to the rest of the cohort. The ratio of temporal to extra-temporal seizure foci in responders was similar to other that of other patients (Fisher exact test: *p* = 0.68), and while there appeared to be a difference in the pathology of resected responders favoring gliosis, this was not significant in comparison to the rest of the cohort (Fisher exact test: *p* = 0.14). Despite lacking a clinical factor to differentiate this group from the rest of the population, based on our results, we estimate the relative proportion of responders in a given population is 19–55% of patients (95% binomial confidence interval with a test sample of 10/27), which demonstrates that patients with potential for significant HFO rate predictive performance could comprise a substantial portion of a large clinical cohort.

## Discussion

In this first-of-its-kind study, we combined advanced automated HFO detection with the intracranial data of a large clinical cohort to investigate the potential use of high-frequency oscillations in seizure prediction. Across patients, we found a significant variation in the ability of time-varying properties of HFO rate to discern a preictal state. After applying a statistical benchmark to the average predictive performance of all models across our cohort, a subset of patient responders was identified that had consistent predictive performance better than random chance. The identification of these 10 individuals represents a novel finding and is our study's most important result. It provides firm support that high-frequency oscillations can function as a temporal biomarker of seizure onset and additionally gives preliminary evidence that seizure prediction using HFOs is not only possible in a clinical context; it can hold significant potential for certain patients.

Another important outcome is the identification of *which* HFO rate features are the most useful. Ranked by their frequency in responder models across multiple windows of time, the most important predictive features of HFO rate included linear slope, variance, and the first quartile cHFO rate within the feature window. The most common feature was the linear slope, which measures gradual changes in HFO rate (either increasing or decreasing), suggesting that these changes are centrally important in determining if a seizure is imminent. One surprising finding was that even HFOs outside the SOZ were useful features. Note that it is not possible to compare relative magnitude of these feature coefficients directly because of the considerable model variability between patients, feature windows, and cross-validation runs. We analyzed the 10 responders and found that three of them had clinical situations in which the OUT channels were likely to be pathological. One patient had a known secondary seizure focus not included in the official SOZ (UMHS-0026), while another had high HFO activity in a non-resected hippocampus that was likely dual pathology from a parietal lesion (UMHS-0040). However, the OUT features were not restricted just to those patients, and thus our finding of predictive value of HFO features outside the SOZ is an intriguing finding. This result suggests that HFOs even outside the SOZ provide important information on identifying impending seizures.

The test AUC values of responder patients we report are within the ranges presented in several seizure prediction studies, notably Brinkmann et al. (2016), Karoly et al. (2017), and Kuhlmann et al. (2018b). There is one caveat to using the AUC metric in seizure prediction, as the inherent imbalance of interictal and preictal data can increase the reported specificity. In order to compare our work with other studies, however, this was an acceptable limitation for our analysis. While no prior work has evaluated HFOs for seizure prediction, there is evidence for a “preictal state” (Stacey et al., 2011). HFOs have been shown to have different signal features (Pearce et al., 2013; Bandarabadi et al., 2019) and changes in rate 30 min before seizures (Scott et al., 2020). Further, some studies have shown distinct changes in high-frequency activity preceding seizure onset; some have also suggested that HFOs could be linked to seizure-generating mechanisms (Worrell et al., 2004).

Despite our positive result, it must be noted that our overall methodology has a number of inherent constraints that limit our findings from being more widely applicable to seizure prediction in general. First, this analysis was based upon processing several minutes of data at a time (10, 15, or 30 min) rather than analyzing features of individual HFOs. There are a wide range of HFO features that could be incorporated into future prediction algorithms. Next, we note that “true” seizure prediction would involve choosing a specific algorithm and testing accuracy prospectively, which was not done here. Second, this method requires HFOs to be present and enough seizures to develop a predictive model; five of our cohort of 32 did not meet this standard. Finally, as stated before these data are limited to only 2 weeks immediately postoperatively during varied medication changes, which is known to be insufficient to have consistent EEG signals and sometimes even atypical seizures. Several of our patients had inconsistent results, but with so few seizures it is impossible to predict whether this would stabilize to an effective solution with more data. A much longer dataset under standard living conditions would be necessary to develop robust algorithms, but such data are not physically possible at present. Future work with a larger dataset could also incorporate additional features of the HFOs themselves (e.g., signal features such as frequency data), as well as previous prediction algorithms using standard EEG. This type of synergistic analysis on larger datasets could have much greater chance at a clinically realizable seizure prediction algorithm.

## Conclusion

Our results show that HFOs can function as a temporal biomarker of seizure onset. We show that changes in the HFO rate are capable of identifying the preictal state up to 30 min before a seizure in some patients. As a preliminary study, our findings are a foundation for future work pursuing individualized seizure-specific prediction efforts, which we envision could eventually function as a tool inside advanced implanted neuromodulation devices that utilize patient-specific and seizure-specific prediction methodologies. Advancement of this HFO seizure prediction framework, however, will require the availability of many chronic high-sampling rate intracranial recordings. While this technology does not yet exist, recent technological improvements have brought it closer to realization—which is sufficient impetus to further investigate HFOs both as a temporal biomarker of epilepsy, and as a potentially powerful predictor of epileptic seizures.

## Data Availability Statement

The raw data supporting the conclusions of this article will be made available by the authors, without undue reservation.

## Ethics Statement

The studies involving human participants were reviewed and approved by University of Michigan IRB. Written informed consent to participate in this study was provided by the participants' legal guardian/next of kin.

## Author Contributions

JS, SG, LK, and WS designed the study and wrote the paper. JS, SG, and WS performed the analyses. SG and WS were responsible for funding. All authors contributed to the article and approved the submitted version.

## Conflict of Interest

WS and SG have a licensing agreement with Natus Medical, Inc. Natus had no involvement in the study. The remaining authors declare that the research was conducted in the absence of any commercial or financial relationships that could be construed as a potential conflict of interest.
